# Knowledge and Practice of PHC Physicians toward the Detection and Management of Hypertension and Other CVD Risk Factors in Egypt

**DOI:** 10.4061/2011/983869

**Published:** 2011-08-11

**Authors:** Mostafa A. Abolfotouh, Laila A. Soliman, Sameh M. Abolfotouh, Mohamed Raafat

**Affiliations:** ^1^Biobanking Section, King Abdullah International Medical Research Center, King Saud Bin-Abdulaziz University for Health Sciences, P.O. Box 22490, Riyadh 11426, Saudi Arabia; ^2^Primary Health Care Department, Ministry of Health and Population, Cairo 21526, Egypt; ^3^Strengthening Rural Health Services Project, Ministry of Health and Population, Cairo 21526, Egypt

## Abstract

*Aim*. To assess the knowledge and practice of PHC physicians toward the detection and management of hypertension (HTN) and other CVD risk factors. *Methods*. A cross-sectional study of all primary health care physicians of the FHU of three rural districts of Egypt was conducted. Each physician was subjected to a prevalidated interview questionnaire on the WHO-CVD risk management package for low and medium resources, and a checklist of observation of daily practices. *Results*. Hypertension was a priority problem in about two-thirds (62.9%) of physicians, yet only 19% have guidelines for HTN patients. Clinical history recording system for HNT was available for 50% of physicians. Levels of knowledge varied with regard to definition of HTN (61.3%, fair), procedures for BP measurement (43.5%, poor), indications for referral (43.5%, poor), patient counseling (61.3%, fair), patient treatment (59.8%, fair). Availability of clinical history recording system for HNT was a significant predictor for physician's level of knowledge (*P* = 0.001). Overall level of practice was fair (68.5%). *Conclusion*. PHC physicians have unsatisfactory knowledge and practice on hypertension. There is a need of more continuing medical education. Local and international manuals, workshops, and seminars on how to make use of these guidelines would improve doctors' performance.

## 1. Introduction

Hypertension is confirmed to be a major health problem in Egypt with a prevalence rate of 26.3% among the adult population (>25 years) [1], with the highest prevalence in greater Cairo (31%) and Northern Upper Egypt (30.7%) and the lowest rate in the frontiers governorates (19.9%). Almost two-thirds (62.5%) of those who could be classified as hypertensive are not aware that they have high blood pressure (BP). At national level, the estimated percentage of hypertensive individuals receiving pharmacological treatment in Egypt was 23.9%, but the hypertension was controlled in only 8% [2].

In this context, hypertension presents a major area of intervention because it is a frequent condition and is amenable to control through both nonpharmacological lifestyle factors and pharmacological treatment. Pharmacological treatment for hypertension has been shown to be effective in decreasing BP and subsequently cardiovascular events [3] although BP levels achieved in treated patients may still be considerably higher than those in truly normotensive persons. Lifestyle measures for lowering BP include reduced alcohol intake, reduced sodium chloride intake, increased physical activity, and control of overweight [4–8]. Lifestyle interventions also have the potential to reduce the need for or the amount of medications in hypertensives and prevent high BP from developing in nonhypertensives. Furthermore, lifestyle interventions are instrumental in controlling other concomitant cardiovascular risk factors not necessarily related to hypertension, such as smoking, raised cholesterol level, or diabetes, hence the importance of a multifactorial approach to effective risk reduction in hypertensives [9–13].

Conventional management of hypertension leaves patients at an unacceptably high risk of cardiovascular events, due to suboptimal blood pressure control and failure to address coexistent risk factors [14, 15]. CVD prevention too frequently focuses on single risk factor rather than on comprehensive cardiovascular risks; therefore, a comprehensive cardiovascular risk management approach is required [16]. CVDs risk factors such as hypertension, diabetes, smoking, high blood lipids, physical inactivity, obesity, and a positive family history often occur together and need to be treated in a comprehensive manner [17].

Despite the resource constraints of health systems, cardiovascular risk management can be addressed, at least in part, through a package of tools that explicitly addresses issues of affordability and that are tailored to suit different levels of health infrastructure. The package is based on best available scientific evidence and takes into consideration the feasibility of applying this evidence in practice. It has been designed for the management of cardiovascular risk in individuals with elevated blood pressure, detected through opportunistic screening. Although it has been primarily designed with hypertension as an entry point, it can be adapted to diabetes or smoking as entry points. The pragmatic approach used offers sufficient flexibility for the package to be applied across all levels of care [16].

A proper assessment and understanding of knowledge, attitude, and practice (KAP) factors among health providers is particularly helpful in the area of chronic conditions such as hypertension, for which prevention and control necessitate a lifelong adoption of healthy lifestyles, as advised by the health providers [18]. Within the health sector reform process at primary level, the Egyptian Ministry of Health and Population (MoHP) is considering the adoption of the WHO-CVD risk management package for low- and medium-resources setting. Before proceeding, the MoHP considers relevant an assessment of knowledge and practice of family practitioners with regards to the management of hypertension and other CVD risk factors. This will assist the MoHP stakeholders in defining the priority content of training courses for the orientation of the health personnel and to adjust the WHO risk management package to the Egyptian situation. Furthermore, the exercise will be helpful in formulating the FHUs and district plan of action and monitoring the progress in this sector during the coming years.

### 1.1. Aim of Study

The aim of this study was to assess the knowledge and practice of PHC physicians toward the detection and management of hypertension and other CVD risk factors.

## 2. Material and Methods

### 2.1. Study Setting

The study was conducted in three rural districts of Egypt; one in the Nile delta (Delengat district, Behaira governorate), and two in Upper Egypt (Armant and Deshna districts, Qena governorate).

### 2.2. Study Design

A cross-sectional study was conducted.

### 2.3. Study Population and Sampling Technique

Two governorates were selected randomly to represent both upper (Qena governorate) and lower (Behaira governorate) Egypt. Then, using the proportionate allocation method of sampling, three districts were selected, one representing Behaira governorate (Delengat district), and two representing Qena governorate (Armant and Deshna districts). All PHC physicians of the Family Health Units (FHUs) of Delengat, Deshna, and Armant districts were included in the study.

### 2.4. Methods

All physicians were subjected to the following techniques.

**(1) An Interview Questionnaire**

The knowledge of PHC physicians was tested by an anonymous questionnaire with close- and open-ended questions, referring to the relevant points on the WHO-CVD risk management package for low and medium resources [16] in a setting where there is a general practitioner. The questionnaire was directly submitted to the PHC physician by a trained interviewer who read the questions and recorded the answers. This questionnaire was constructed to collect data of Knowledge on (1) definition and diagnosis of hypertension, (2) patient history (heart attack, angina, chest pain, stroke, TIA, cardiac failure, PVD, family history, tobacco use, alcohol intake, medication, and drug use), (3) risk factors to determine (gender, age, smoking, family history, obesity, diabetes, and stress), (4) what needs to evaluate if patient has hypertension (body weight, features of secondary HNT, evidence of cardiac failure, check urine sugar and albumen, and ECG), (5) indications for referral (severe hypertension, history of heart attack/angina/TIA, stroke, heart failure, diabetes, hypertensive complications/emergency, secondary hypertension, pregnancy, positive urine albumen or glucose in undiagnosed diabetic case, and hypertensive retinopathy), (6) advices given to patients (dietary advices and the role of nurse in patient counseling), and (7) patient treatment (when to start treatment, choice of drugs, and definition of complicated hypertension).

The interview was pretested and assessed for validity (based upon a panel of experts in hypertension), and test-retest reliability was applied on 5 subjects, with Cronbach alpha 0.87. The number of items in agreement with the WHO risk management package was used as a measure of knowledge.

A scoring system was applied by adding 1 point to each correct answer, and 0 point to each wrong answer. Then a total score was estimated for every knowledge item, and a percentage mean score was calculated. Level of knowledge was considered poor if the percentage mean score was less than 50%, fair if from 50 to less than 75%, and good if more than 75%.

**(2) An Observation Checklist**

The practice was assessed by direct observation of daily activities during the conduction of outpatient visits, especially if a new hypertension case was detected. This direct observation was facilitated by using a specific checklist to observe physicians when managing only patients attending OPD and aged >25 years (*n* = 43). The check list included general and specific procedures of BP measurement, and the equipment used. A scoring system was applied by adding 1 point to each correct practice, and 0 point to each wrong practice. Then a total score was estimated for practice items, and a percentage mean score was calculated. Level of practice was considered poor if the percentage mean score was less than 60%, fair if from 60 to 80%, and good if more than 80%.

### 2.5. Data Analysis

Statistical analysis was done with aid of the computer program SPSS (statistical package for the social sciences). Descriptive measures as arithmetic mean and standard deviation were used to describe quantitative data, and proportions and their corresponding 95% confidence intervals were used for qualitative data. Mann-Whitney test and Kruskal Wallis test were used to compare between sample means for quantitative data. Multiple linear regression analysis was used to assess the relationship between the different body composition measures and level of knowledge, adjusting for age, sex, years of experience, availability of guidelines, and record system. Statistical significance was set at *P* < 0.05.

## 3. Results and Discussion

There was a total of 62 PHC physicians. Their ages ranged from 25 to 59 years with a mean age of 34.6 and a standard deviation (SD) of 9.1 years. Eighty-seven percent of the PHC physicians were males and only 14 (22.6%) have postgraduate qualifications in the form of diploma, master, and board. Their years of experience ranged from one year to 31 years, with a mean duration of 8.7 ± 8.3 years. For more than half of the physicians, the years of experience ranged from 1 to 5 years, while those with more than 10 years constituted 32% of all physicians (Table 1).

Without a systematic attempt to screen and follow up our patients and to audit this process, the “rule of halves” will apply. This rule implies that half of the hypertensive patients will be undiscovered, half of them will be treated, and only half of those receiving treatment will be adequately controlled [19]. In the present study, hypertension was considered a priority problem in the FHU catchment area by about two-thirds (62.9%) of physicians, yet only 19% have guidelines for HBP patients. Clinical history recording system for HBP was available for 50% of physicians; however, only 30.6% of all physicians use this system regularly. The majority of physicians (79%) reported that more than 60% of their patients were controlled (Table 1). However, it seems that physicians overreported control of blood pressure compared to national figures based on actual measurements [2]. 

Table 2 shows the accuracy of the knowledge of PHC physicians in detection and management of HNT. A total of 38 physicians (61.3%) correctly defined HTN. This finding was not in agreement with the finding of another study in Saudi Arabia [20], where only a low percentage of PHC physicians knew the correct definition. However, those who agreed upon the necessity of measurement recheck after 5–10 minutes rest were only 4 (6.5%) physicians. Levels of knowledge in the present study varied with regard to definition of HTN (61.3%, fair), procedures for BP measurement (43.5%, poor), what to evaluate? (45.2%), indications for referral (43.5%, poor), patient counseling (61.3%, fair), and patient treatment (59.5%, fair).

Measurement of BP can detect HTN even in the early presymptomatic phase, and the screening technique is simple, cheap, and acceptable. The initial hypothesis was that screening for HTN was not carried out appropriately in primary health care (PHC) services. This was inferred from studies carried out on hypertension [21], and other chronic illnesses such as diabetes mellitus [22], bronchial asthma [23], and epilepsy [24]. These studies have collectively concluded that the chronic diseases were not adequately screened for, and patients were not appropriately controlled. However, the present study revealed that BP was routinely taken for patients in all except one family health unit (FHU), yet performance, generally, was unsatisfactory where most of the items of the procedure of measurement were not fulfilled in many of those units (Table 3). The degree of coverage of performance of specific tasks applied to patients during BP measurements varied from 55.8% for the sitting positioning of patients, to 98% applying the cuff directly on the skin. All the items pertained to the sphygmomanometer were covered correctly in nearly all the units. Regarding the measurement procedure, the degree of coverage of different tasks was generally not satisfactory. It varied from <5% (for explanation of the procedure to the patient) to 67.4% (for positioning the stethoscope and palpating the radial pulse), to 79% (for resting the patient before measurement). The overall level of practice of PHC physicians on blood pressure measurement was fair (68.5%, 95% CI = 57.4 : 79.6). 

Table 4 shows the percentage mean score of knowledge on hypertension in relation to some physicians' characteristics. Availability of patient clinical history recording system was the only variable significantly associated with the physicians' level of knowledge. Even after adjustment for all other confounders, this association remained significant, with a higher level of knowledge—as estimated by the percentage mean knowledge score—when this recording system is available. It has been reported that doctor's knowledge is reduced after 10 years of experience [25]. However, this was not the case in the present study, where experience was not significantly associated with the percentage mean knowledge score. This might reflect the necessity of continuing medical education programs for all physicians irrespective of their previous experience. 

This study has some limitations which should be considered when interpreting these findings. First, the study relied upon a written questionnaire for information on knowledge and attitude on management of hypertension, and this tool might have affected the validity of information collected. Second, we cannot be certain of the causal direction of the association observed between the level of knowledge and some physicians' characteristics, due to the study's cross-sectional design. Third, the control of blood pressure based on the physician reports is subjected to information bias, and it seems that physicians overreport control of blood pressure compared to national figures based on actual measurements.

In conclusion, the PHC physicians in the study areas have unsatisfactory knowledge and practice on hypertension. Availability of patient clinical history recording system was the only variable significantly associated with the physicians' level of knowledge. It is evident that physicians are in need of more continuing medical education to improve their knowledge, and practice toward HTN. Available local and international manuals should be made accessible to PHC, and workshops and seminars on how to make use of these guidelines would improve doctors' performance.

## Figures and Tables

**Table 1:** Demographic and other characteristics of 62 PHC physicians.

Characteristics	No.	%
Physician's sex		
Male	54	87.1
Female	8	12.9
Physician age		
<30	30	48.4
30−	23	37.1
45−	9	14.5
Years of experience		
1–5	35	56.5
6–10	7	11.2
>10	20	32.3
Is hypertension a priority problem in your catchment population? (yes)		
Yes	39	62.9
No	19	30.6
Do not know	4	6.5
Availability of guidelines for hypertension patient?	12	19.4
Availability of patient clinical history recording system	31	50.0
Percentage of your patients with hypertension is under control?		
<40%	2	3.2
40–60%	5	8.1
>60%	49	79.0
Do not know	6	9.7

*Percentages denote “yes” answer to each statement.

**Table 2:** The accuracy of the knowledge of 62 PHC physicians on detection and management of hypertension.

Knowledge categories	No.	%	95% CI
(1) Definition of hypertension	38	61.3	49.2 : 73.4
(2) Procedures before and after BP measurement	27	43.5	31.2 : 55.8
(3) Patient's history taking (risk factors)	27	43.5	31.2 : 55.8
(4) What to evaluate?	28	45.2	32.8 : 57.6
(5) Indications for referral	27	43.5	31.2 : 55.8
(6) Patient counseling:	38	61.3	48.9 : 72.3
(a) dietary advice	43	69.4	57.9 : 80.9
(b) role of FUH nurse in patient counseling	16	25.8	14.9 : 36.7
(7) Patient treatment:	37	59.7	47.5 : 71.9
(a) when to start treatment	54	87.1	78.8 : 95.4
(b) choice of drugs	39	62.9	50.9 : 74.9
(c) definition of uncomplicated hypertension	58	93.5	87.4 : 99.6

Overall knowledge percentage score	62	51.3	38.9 : 63.7

*Percentages denote correct answers to each category.

**Table 3:** Results of 43 PHC physicians CVD observation checklist for outpatient department.

	No. (*n* = 43)	%	95% CI
General procedures			
General physical examination done	28	65.1	(53.2 : 77.0)
Blood pressure taken	42	97.7	(94.0 : 101.4)

The patient:			
Sitting Position	24	55.8	(43.4 : 68.2)
Arm supported on desk, pillow, and so forth	32	74.4	(63.5 : 85.3)
Midpoint of supper arm at level of the heart	33	76.7	(66.2 : 87.2)
Tight sleeves and/or collars removed	29	67.4	(55.7 : 79.1)
Sphygmomanometer cuff applied directly on the skin	42	97.7	(94.0 : 101.4)

Equipment			
What mercury sphygmomanometer	34	79.1	(69.0 : 89.2)
Aneroid	9	20.9	(10.8 : 31.0)
With deflated cuff the manometer read zero	43	100	—
Rubber bladder inside cuff around 40% of the upper arm circumference	42	97.7	(94 : 101.4)
Sphygmomanometer valve working properly (open and closing)	43	100	—

Measurement procedure			
Patient rested for at least 5 minutes	34	79.1	(69 : 89.2)
BP procedure explained	2	4.7	(−0.6 : 10)
Lower end of the cuff one inch above the antecubital fossa	28	65.1	(53.2 : 77.0)
Index and middle fingers palpate the radial pulse	29	67.4	(56.0 : 79.1)
Stethoscope applied over brachial artery, but not touching tubing or the cuff	29	67.4	(56.0 : 79.1)
Blood pressure measurement in both arms	7	16.3	(7.1 : 25.5)
More than one blood pressure measurement few minutes apart	11	25.6	(14.7 : 36.5)

Overall correct practice (%)	43	68.5	(57.4 : 79.6)

*Percentages denote correct practices.

**Table 4:** Percentage mean score of knowledge on hypertension in relation to some physicians' characteristics.

Characteristics	Knowledge score %			
	Mean	SD	statistical difference	Adj. *P* value
Physician's sex				
Male	52.40	13.26	Z = 1.61	0.16
Female	42.86	17.00	*P* = 0.11	
Physician age				
<30	52.22	15.59	KW = 1.13	0.12
30−	51.61	13.56	*P* = 0.57	
45−	47.42	8.75		
Years of experience				
1–5	50.76	15.19	KW = 0.09	0.12
6–10	50.77	17.88	*P* = 0.96	
>10	52.35	10.35		
Having specialty				
Yes	49.36	10.61	*Z* = 0.84	0.80
No	51.87	14.87	*P* = 0.40	
Availability of guidelines for hypertension patient?				
yes	50.45	10.47	*Z* = 0.45	0.18
No	51.48	14.78	*P* = 0.65	
Availability of patient clinical history recording system	31	50.0		
Yes	57.53	12.58	*Z* = 3.34	0.001*
No	45.62	12.78	*P* = 0.001*	

*Z*—Mann-Whitney test was applied, and KW—Kruskal Wallis test was applied. SD—standard deviation, *statistical significance.

## Abbreviations

PHC: Primary health care
FHU: Family health unit
HNT: Hypertension.
